# Development of a New DNA Vaccine for Alzheimer Disease Targeting a Wide Range of Aβ Species and Amyloidogenic Peptides

**DOI:** 10.1371/journal.pone.0075203

**Published:** 2013-09-27

**Authors:** Yoh Matsumoto, Naoko Niimi, Kuniko Kohyama

**Affiliations:** 1 Department of Molecular Neuropathology, Tokyo Metropolitan Institute for Neuroscience, Tokyo, Japan; 2 Department of Immunotherapy Development, Tokyo Metropolitan Institute of Medical Science, Tokyo, Japan; Glaxo Smith Kline, Denmark

## Abstract

It has recently been determined that not only Aβ oligomers, but also other Aβ species and amyloidogenic peptides are neurotoxic in Alzheimer disease (AD) and play a pivotal role in AD pathogenesis. In the present study, we attempted to develop new DNA vaccines targeting a wide range of Aβ species. For this purpose, we first performed *in vitro* assays with newly developed vaccines to evaluate Aβ production and Aβ secretion abilities and then chose an IgL-Aβx4-Fc-IL-4 vaccine (designated YM3711) for further studies. YM3711 was vaccinated to mice, rabbits and monkeys to evaluate anti-Aβ species antibody-producing ability and Aβ reduction effects. It was found that YM3711 vaccination induced significantly higher levels of antibodies not only to Aβ1-42 but also to AD-related molecules including AβpE3-42, Aβ oligomers and Aβ fibrils. Importantly, YM3711 significantly reduced these Aβ species in the brain of model mice. Binding and competition assays using translated YM3711 protein products clearly demonstrated that a large part of antibodies induced by YM3711 vaccination are directed at conformational epitopes of the Aβ complex and oligomers. Taken together, we demonstrate that YM3711 is a powerful DNA vaccine targeting a wide range of AD-related molecules and is worth examining in preclinical and clinical trials.

## Introduction

Alzheimer’s disease (AD) is the most common cause of age-related cognitive decline, affecting more than 12 million people worldwide. The disease is characterized by progressive memory impairment, cognitive decline, altered behavior and language deficit. Later, patients show global amnesia and slowing of motor function, and finally death [1]. It is generally believed that accumulation of amyloid beta (Aβ) is the first event in the pathogenesis of AD, followed by tau phosphorylation, tangle formation and neuronal death (amyloid cascade hypothesis) [2], [3]. Therefore, deposited or depositing Aβ should be the first target of AD therapy.

Recently, several immunotherapies have been developed as curative treatments of AD by targeting the underlying cause. In 1999, Schenk and his colleagues demonstrated that monthly inoculation with synthetic Aβ in adjuvants could lead to high anti-Aβ antibody titers and dramatic reductions of Aβ deposition in PDAPP transgenic mice [4]. Subsequent studies demonstrated that clearance of Aβ deposits following immunization protected amyloid precursor protein (APP)-transgenic mice from developing memory deficits [5], [6]. Approximately 50% reduction in Aβ plaques is sufficient to ameliorate cognition [7]. Based on the promising results using model mice, clinical trials with an Aβ peptide vaccine, AN1792, were started. However, a phase II-A study was halted due to the development of meningoencephalitis in 18 of 298 patients (6%) who received the vaccine [8].

The outcome of Aβ immunotherapies is controversial. Autopsy of an AN1792-treated patient revealed a significant reduction of Aβ plaques compared with unimmunized patients [9]. However, Holms et al. reported later that although AN1792 immunization resulted in clearance of Aβ plaques, this clearance did not prevent progressive neurodegeneration [10]. Since these studies were performed in a relatively small scale and the number of autopsied patients was too small, it is essential to obtain more information to draw final conclusion. Recently, it was reported that AN1792 immunization provided beneficial effects on neurite morphology and tau pathology [11]. Furthermore, clinical trials with a humanized anti-Aβ monoclonal antibody, Bapineuzumab, revealed that the treatment improved cognitive decline and retarded the brain volume loss in APOE4 non-carrier patients [12].

Progress in understanding pathomechanisms of AD revealed that not only Aβ1-42, but also other Aβ species and amyloidogenic peptides that have no amino acid homology to Aβ are involved in neurotoxicity in the brain [13]. Based on such information, the present study was undertaken to develop DNA vaccines targeting a wide range of Aβ species and amyloidogenic peptides and succeeded in reducing Aβ and Aβ species with a newly developed DNA vaccine, YM3711.

## Results

### Aβ Tetramer Structure and Addition of Immunoglobulin Fc Portion Upregulate Aβ-protein Complex Production and its Secretion into the Extracellular Space

**Figure 1:**
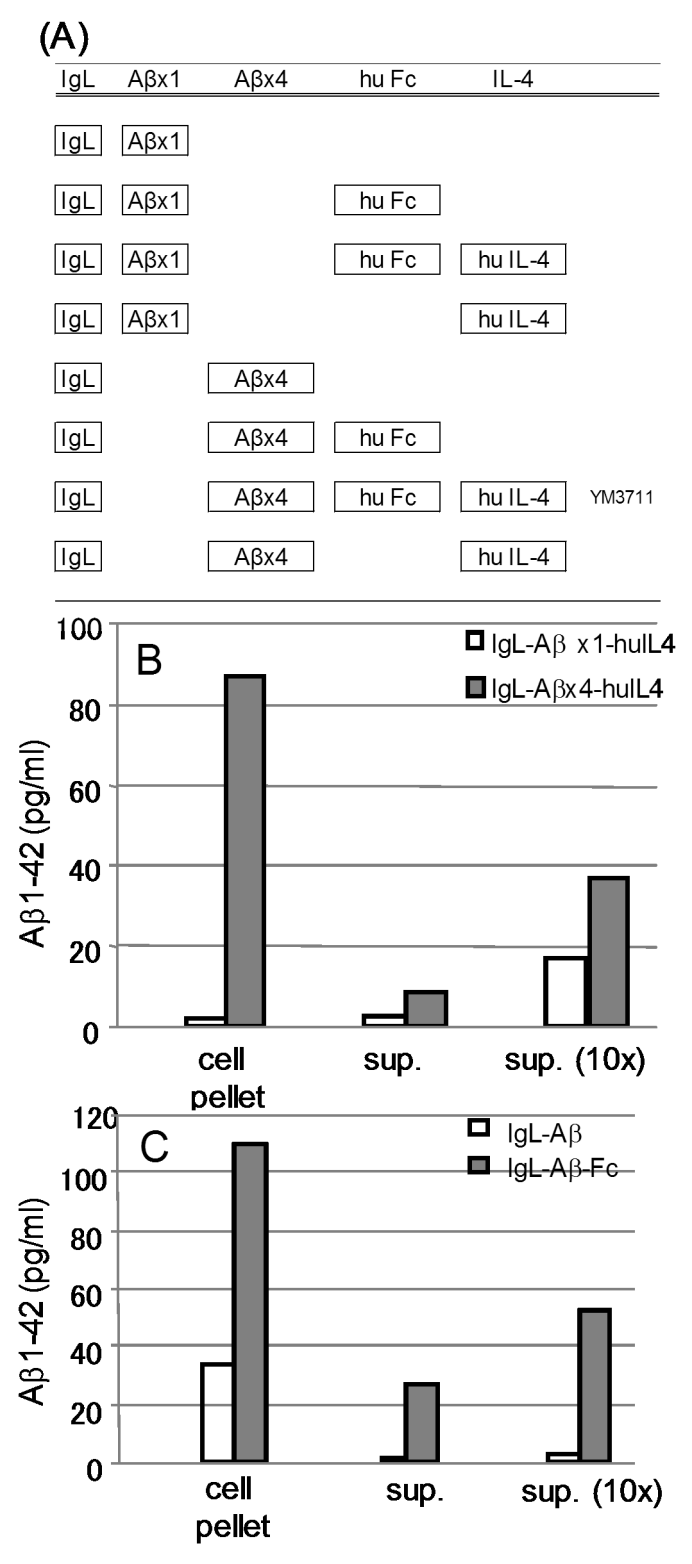
DNA vaccines prepared and examined in the present study (A) and effects of the tandem structure (B) and addition of Fc (C) in vitro potency assay. (**A**) Aβx4, four-repeated Aβ1-42 sequence; hu Fc, human Fc portion of immunoglobulin; hu IL-4, human IL-4 sequence. (**B** and **C**) HEK293 cells were cultured and transfected with the indicated DNA vaccines. The supernatant and cell pellet of transfected cells were harvested and subjected to Aβ quantitation using the Human β Amyloid (1-42) ELISA Kit *Wako*, High-Sensitive according to the manufacturer’s instruction. Sup and sup (10×) indicate unconcentrated and concentrated supernatant, respectively. Mean values of triplicate assay are shown.

It has recently been determined that in Alzheimer disease, not only Aβ dimers and oligomers, but also posttranslationally modified Aβ species and other amyloidogenic peptides are neurotoxic [14], [15]. In the present study, we attempted to develop new DNA vaccines targeting these molecules. For this purpose, we employed the tandem-repeats of Aβ to increase the antibody production ability and to induce wide-range antibodies for Aβ species. Addition of the Fc portion of immunoglobulin and IL-4 was performed to upregulate the extracellular movement of the Aβ-Fc complex and to upregulate antibody production, respectively (Fig. 1A).

The indicated vaccines were transfected to cultured cells and the amount of Aβ in the supernatant and cell pellet were determined by a sandwich ELISA (Fig. 1B and 1C). It was clearly demonstrated that Aβ tetramer structure (Fig. 1B) and addition of immunoglobulin Fc portion (Fig. 1C) upregulated Aβ-protein complex production (cell pellet) and its secretion into the extracellular space (sup). Based on these results, we chose IgL-Aβx4-huFc-huIL-4 (Fig. 1A, code, YM3711) for further experiments.

**Figure 2:**
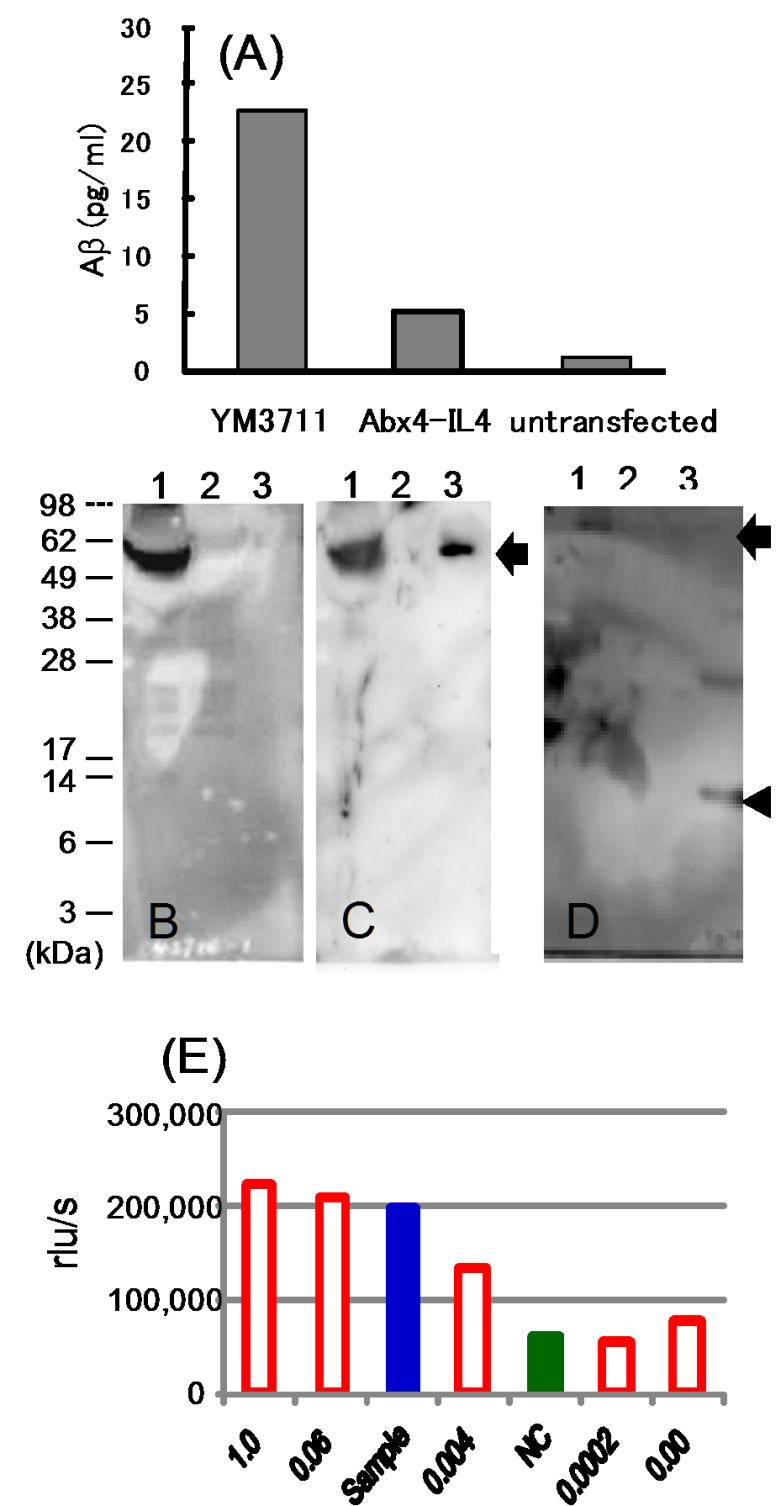
*In vitro* characterization of YM3711. (**A**) Quantitation of Aβ production in the culture supernatant of YM3711- or Aβx1-IL-4-transfected cells by sandwich ELISA. Mean values of triplicate assay are shown. (**B–D**) Western blot analysis of translated YM3711 products. YM3711 was transfected into cultured cells and culture supernatant was harvested, electrophoresed, blotted and immunostained with anti-Aβ (B), anti-Fc (C) and anti-IL-4 (D) antibodies. Lane 1, supernatant of YM3711-transfected cells; lane 2, supernatant of untransfected cells; lane 3, positive control (B, synthetic Aβ1-42 peptide; C, human Fc portion; D, recombinant human IL-4). Immunostaining for Aβ and Fc revealed one band (ca 58 kDa) (B and C). IL-4 staining also revealed one band (ca 58 kDa) in the supernatant of YM3711-transfected cells (an arrow in lanes 1), but not in the supernatant of untransfected cells (lane 2) corresponding to the band for recombinant IL-4 (ca 14 kDa) (lane 3, arrowhead). The upper band in lane 3 was judged to be a dimer according to its molecular size (ca 28 kDa). (**E**) IL-4 bioassay of the supernatant of YM-3711-transfected cells. IL-4 activities were determined by bioassay using an IL-4-dependent cell line, TF-1. The supernatant of YM3711-transfected cells contained active IL-4 compatible to 0.06 ng/ml human recombinant IL-4 (blue bar, sample). The culture supernatant of untransfected culture cells showed IL-4 activities corresponding to 0.0002 ng/ml (green bar, NC). Open red bars indicate serially diluted recombinant IL-4.

We also performed in vitro studies to characterize the nature of YM3711. In the transfection assay, a sufficient amount of the YM3711 product was secreted in the culture medium compared with Aβx1-huIL-4 (Fig. 2A). The product was electrophoresed, blotted and immunostained with anti-Aβ (Fig. 2B), anti-Fc (Fig. 2C) and anti-IL-4 (Fig. 2D). Immunostaining for Aβ and Fc revealed one band for YM3711 products (estimated molecular size, 58 kDa) (lane 1 in Fig. 2B and 2C). IL-4 staining also revealed one band (ca 58 kDa) in the supernatant of YM3711-transfected cells (an arrow in lanes 1 of Fig. 2D), but not in the supernatant of untransfected cells (lane 2), corresponding to the band for recombinant IL-4 (ca 14 kDa) (lane 3, arrowhead). The upper band in lane 3 was judged to be a dimer according to its molecular size (ca 28 kDa). We also evaluated IL-4 bioactivities of the culture supernatant of YM3711-transfected cells using an IL-4-dependent cell line. As shown in Fig. 2E, the supernatant (blue bar, labeled as sample) contained active IL-4 compatible to 0.06 ng/ml recombinant IL-4. Open red bars indicate serial diluted recombinant IL-4. The culture supernatant of untransfected cells showed IL-4 activities corresponding to 0.0002 ng/ml (gree bar, NC). Thus, it was demonstrated that translated YM3711 product showed significant IL-4 bioactivities (Fig. 2E).

### *In vivo* Administration of YM3711 Reduces not only Aβ1-42 but also other Aβ Species in Model Mice

**Figure 3:**
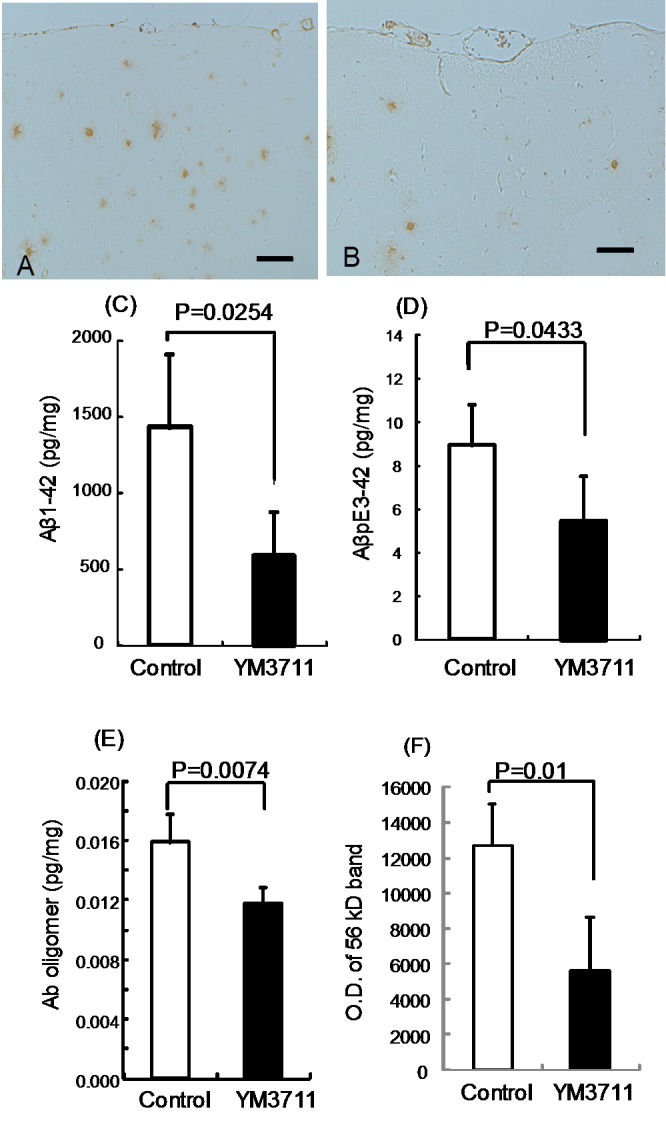
YM3711 vaccination effectively reduced not only Aβ1-42 but also other Aβ species. YM3711 at a dose of 100 µg was injected weekly to 15-month old model mice for 6 weeks and the brain and plasma were taken at 8 weeks. Immunostaining for Aβ revealed that compared with untreated control mice (**A**), Aβ deposits in the frontal cortex were clearly decreased in vaccinated mice (**B**). Bar = 100 µm. Quantitative analysis by sandwich ELISA (**C–E**) demonstrated the significant reduction of Aβ1-42 in treated mice (**C**) (p = 0.0254). AβpE3-42 (**D**) and Aβ oligomer (**E**) quantitation also revealed significant reduction in the YM3711-treated group (p = 0.0433 and p = 0.0074, respectively). Aβ species that are recognized by anti-amyloid fibrils, OC, were semiquantitated by measuring densities of 56 kD band (**F**). Aβ fibrils were significantly reduced in treated Tg mice than in control Tg mice (p = 0.01). Closed and open columns indicate the amount of Aβ species in the cerebral cortex of vaccinated mice (n = 4) and of age-matched control mice (n = 5), respectively.

**Figure 4:**
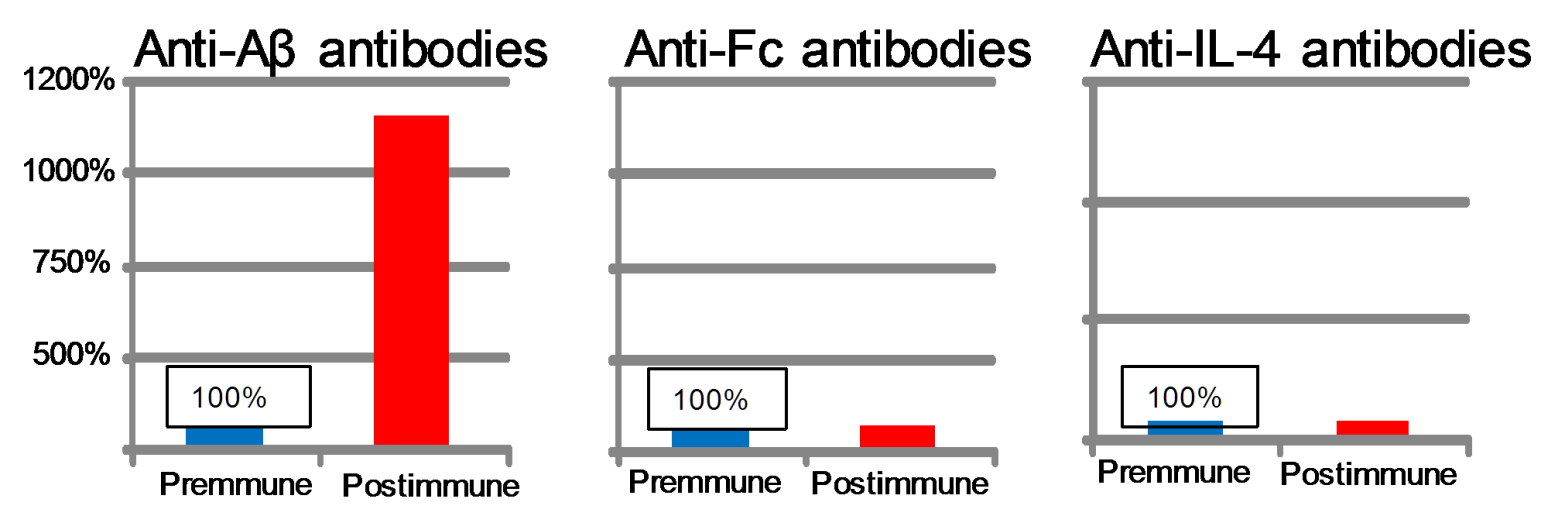
Presence or absence of antibodies against vaccine components by YM3711 vaccination. Monkeys (n = 4) were injected with YM3711 six times and the presence or absence of antibodies against vaccine components such as Aβ, human Fc and human IL-4 was determined. It was clearly demonstrated that YM3711 vaccination induced antibodies against Aβ components vigorously (left panel), while anti-Fc and anti-IL-4 antibodies were not observed in all the samples examined (middle and right panels).

YM3711 at a dose of 100 µg was injected weekly to B6C3-Tg (APPswe, PSEN1dE9) 85Dbo/J mice (hereafter referred to as Tg mice in the present study) for 6 weeks and the brain and plasma were taken at 8 weeks. Immunostaining for Aβ in the brain revealed that compared with untreated control mice (Fig. 3A), Aβ deposits in the frontal cortex were clearly decreased in vaccinated mice (Fig. 3B). Quantitative analysis by sandwich ELISA (Fig. 3C–3F) demonstrated a significant reduction of Aβ1-42 in treated mice (Fig. 4C) (p = 0.0254). Furthermore, AβpE3-42 (Fig. 3D) and Aβ oligomer (Fig. 3E) quantitation revealed significant reduction in the YM3711-treated group (p = 0.0433 and p = 0.0074, respectively). In addition, Aβ species that are recognized by anti-amyloid fibrils, OC, were semiquantitated by measuring densities of 56 kD band (Fig. 3F). It was found that Aβ fibrils were significantly reduced in treated Tg mice than in control Tg mice (p = 0.01).

### YM3711 Vaccination Induced Antibodies against Aβ, but not against other Components of the Vaccine

We first examined the presence or absence of antibodies against vaccine components other than Aβ, i.e. human Fc and IL-4 (Fig. 4). Monkeys were injected with YM3711 six times and the presence or absence of antibodies against vaccine components such as Aβ, human Fc and human IL-4 was examined. As shown in Fig. 4, it was clearly demonstrated that YM3711 vaccination induced antibodies against Aβ components vigorously (Fig. 4, left panel), while anti-Fc and anti-IL-4 antibodies were not observed in all the samples examined (Fig. 4, middle and right panels). This was probably because Fc and IL-4 are present in monkeys for life time and very similar between humans and monkeys in terms of the amino acid sequence. Therefore, monkeys may be in a tolerant state to exogenous human Fc and IL-4.

### YM3711 Induces Antibodies against Aβ Species and Amyloidogenic Peptides

**Figure 5:**
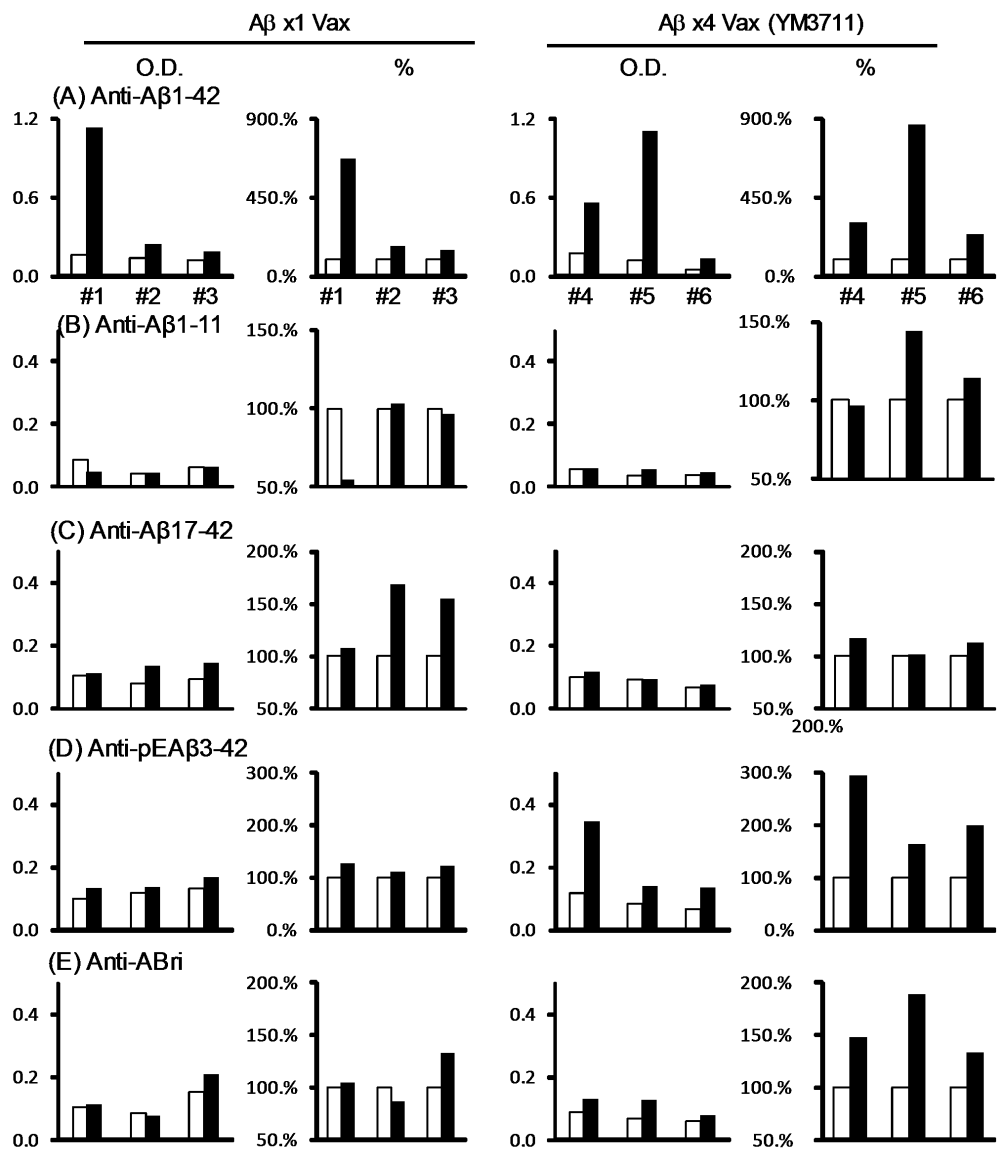
YM3711 vaccination induced antibodies not only to Aβ species but also to an unrelated amyloidogenic peptide. Rabbits (3 rabbits per group) were immunized with either IgL-Aβ-Fc-IL-4 (Ax1 Vax, Rabbits #1, #2 and #3) or IgL-Aβx4-Fc-IL-4 (YM3711, Rabbits #4, #5 and #6) once a week for 6 weeks and preimmune (open bars) and postimmune final (closed bars) plasma was collected and the titers of antibodies against Aβ1-42 (**A**), Aβ1-11 (**B**), Aβ17-42 (**C**), AβpE3-42 (**D**) and ABri (**E**) were determined by ELISA. O.D. values and percent increase of each assay are shown. To avoid inter-assay variations, all the samples to be compared were examined in the same assay.

To establish newly developed therapeutics, it is essential to examine the efficacy and safety of candidate drugs in large animals, such as rabbits and monkeys. This is because drugs that are effective in mice and rats are not always effective in large animals. Rabbits (3 rabbits per group) were immunized with either IgL-Aβ-Fc-IL-4 (Aβx1 Vax, Rabbits #1, #2 and #3) or IgL-Aβx4-Fc-IL-4 (YM3711, Rabbits #4, #5 and #6) once a week for 6 weeks (Fig. 5) and preimmune (open bars) and postimmune final (closed bars) plasma was collected and the titers of antibodies against Aβ1-42 (Fig. 5A), Aβ1-11 (Fig. 5B), Aβ17-42 (Fig. 5C), pyroglutamate-modified Aβ (AβpE3-42) (Fig. 5D) and ABri (Fig. 5E) were determined by ELISA. While anti-Aβ1-42 antibodies were elevated in one of three rabbits that had been injected with IgL-Aβ-Fc-IL-4 vaccine (left half of panel A), all the rabbits immunized with YM3711 (right half of panel A) showed significant increase of the antibodies. Anti-Aβ1-11 antibodies were found only in the YM3711 group (panel B) and the titers of anti-Aβ17-42 were higher in the Aβx1 Vax groups (panel C). It should be noted that antibodies against a posttranslationally modified Aβ species, AβpE3-42, were significantly elevated only in the YM3711 group (panel D). YM3711, but not Aβx1 Vax, induced anti-ABri antibodies (panel E). Similarly, YM3711 vaccination induced high titers of anti-Aβ antibodies in monkeys (data not shown).

**Figure 6:**
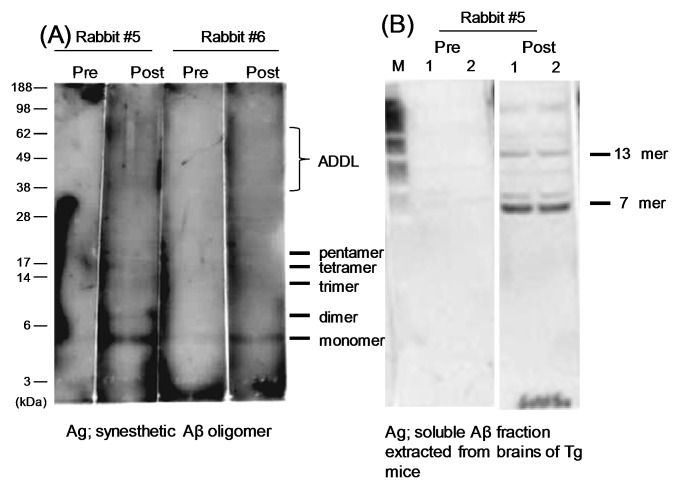
Induction of anti-Aβ oligomer antibodies by YM3711 vaccination. (**A**) In Rabbit #5, anti-Aβ trimer antibodies were detected in postimmune plasma in addition to anti-monomer antibodies. Anti-tetramer and pentamer antibodies became visible after YM3711 vaccination. Antibodies to ADDL (Aβ-derived diffusible ligand) were also detectable. Antibody elevation in Rabbit #6 was slightly weak compared with Rabbit #5 but a similar antibody profile was recognizable (right half). Aβ oligomers were prepared using synthetic peptides. (**B**) Rabbit #5 plasma stained 7mer and 13mer Aβ oligomers. Aβ oligomers were extracted from brain tissues of Tg mice.

We also examined whether YM3711 vaccination induces antibodies against Aβ oligomers (Fig. 6). In Rabbit #5, anti-Aβ trimer antibodies, which detected a 12 kDa band, were evident in postimmune plasma in addition to anti-monomer antibodies, which detected a 4 kDa band. Anti-tetramer and pentamer antibodies became visible after YM3711 vaccination. Antibodies to ADDL (Aβ-derived diffusible ligand) were also detectable. Antibody elevation in Rabbit #6 was slightly weak compared with Rabbit #5 but a similar antibody profile was recognizable (Fig. 6, right half). Similar analysis was made using soluble Aβ extracted from brain tissue of model mice (Fig. 6B). As clearly seen, 7-mer (ca 28 kDa) and 13-mer (ca 52 kDa) Aβ oligomers were stained with vaccinated, but not preimmune, plasma, again indicating that YM3711 induces antibodies against Aβ oligomers. Collectively, YM3711 vaccination induces antibodies not only to a variety of Aβ species but also to an unrelated (in terms of the amino acid sequence) amyloidogenic peptide.

### Characterization of YM3711 Products (YM3711P) and Antibodies Induced by YM3711 Vaccination

**Figure 7:**
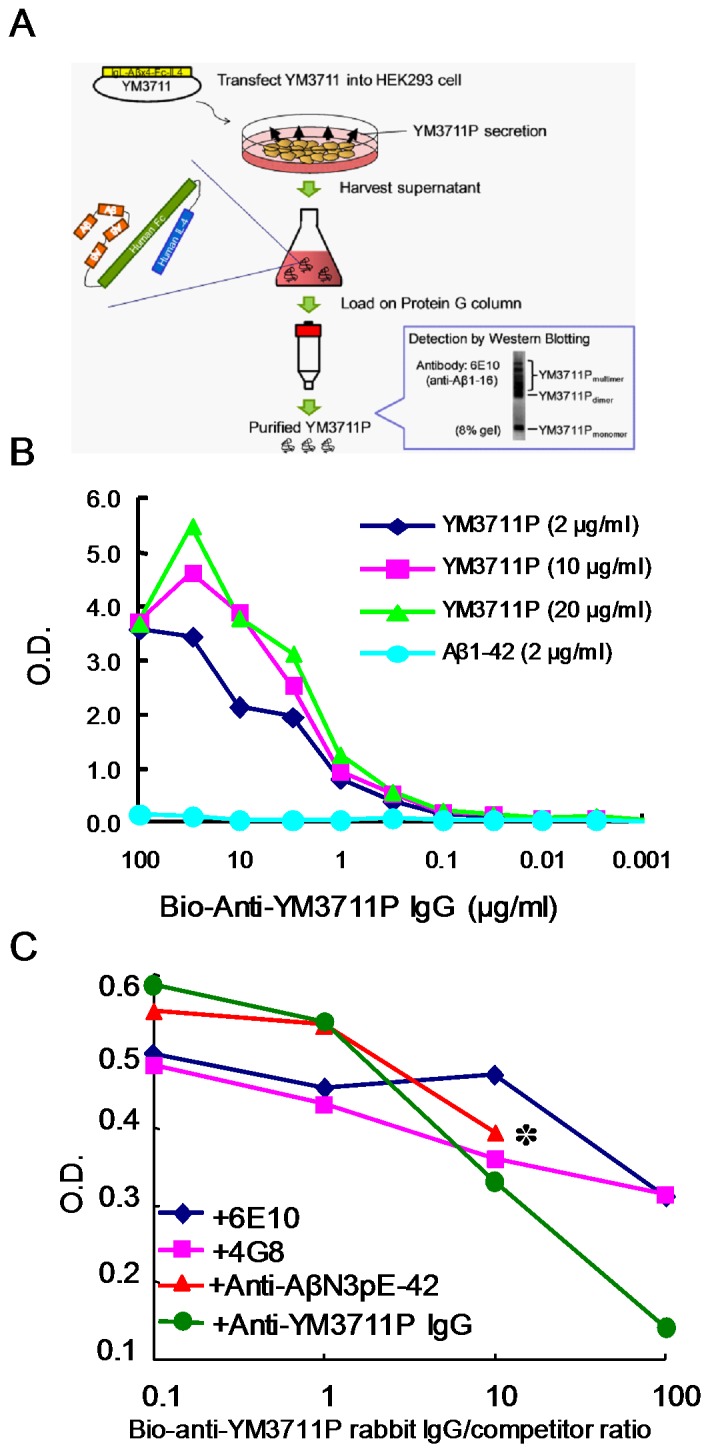
Purification of YM3711 products (YM3711P) (A) and binding (B) and competition (C) assays using YM3711P. (**A**) YM3711 was transfected to floating HEK293 cells using the FreeStyle 293 Expression System. After 4 day culture, the supernatant was harvested and filtered. Then, YM3711P were purified with an affinity column (HiTrap NHS-activated HP column) coupled with anti-Aβ1-42 antibodies. The eluate was evaluated by Western blotting with 6E10 mAb. (**B**) **Binding assay.** YM3711P at concentrations of 2 µg/ml (diamonds), 10 µg/ml (squares) and 20 µg/ml (triangles) or Aβ1-42 (2 µg/ml, circles) were coated onto microtiter wells. After blocking, biotinylated IgG purified from plasma of rabbits that had been vaccinated with YM3711 were applied and followed by HRP-labeled VECTSTAIN Elite ABC Kit. Samples showing O.D. more than 2.5 were further diluted and reexamined. Calculated O.D. values are shown in Panel B. (**C**) **Competition assay.** YM3711P at a concentration of 2 µg/ml was applied onto microtiter wells. Then, wells were incubated with a mixture of biotinylated anti-YM3711P IgG and unlabeled various competitors at 0.1 to 100 ratios. Competitors included 6E10 (diamonds), 4G8 (squares), anti-AβpE3-42 antibodies (triangles) and unlabeled anti-YM3711P IgG (circles). An asterisk indicates that higher concentration of the reagent was not available.

As shown above, YM3711 vaccination induced antibodies not only to Aβ1-42, but also to a variety of Aβ species and amyloidogenic peptides unrelated to Aβ in terms of the amino acid sequence. We hypothesized that antibodies induced by YM3711 vaccination direct not only at Aβ molecules but also at conformational epitopes, which are common to Aβ oligomers and amyloidogenic peptides. To further characterize the nature of YM3711-derived Aβ-protein complex and YM711-induced antibodies, we first purified YM3711 products (YM3711P) (Fig. 7A) and performed binding and competition assays. For the binding assay, YM3211P obtained from the culture supernatant of YM3711-transfected HEK 239 cells were coated onto 96-well plates. Then, biotinylated IgG purified from plasma of rabbits that had been vaccinated with YM3711 and HRP-labeled VECTSTAIN Elite ABC were applied. The results are summarized in Fig. 7B. Purified IgG contained high titers of anti-YM3711P antibodies, whereas anti-Aβ1-42 antibodies were very low. This finding strongly suggests that a large part of antibodies in YM3711-vaccinated rabbits direct at conformational epitopes of the Aβ complex.

Then, we performed a competition assay (Fig. 7C). Microtiter wells coated with YM3711P were incubated with a mixture of biotinylated anti-YM3711P IgG and various unlabeled competitors at 0.1 to 100 ratios. As expected, cold anti-YM3711 IgG showed strong competing abilities in a dose-dependent manner, whereas two anti-Aβ mAbs and anti-AβpE3-42 antibodies exhibited relatively weak competing abilities.

### Antibodies Raised by YM3711 Vaccination Bind to Amyloid Plaques

**Figure 8:**
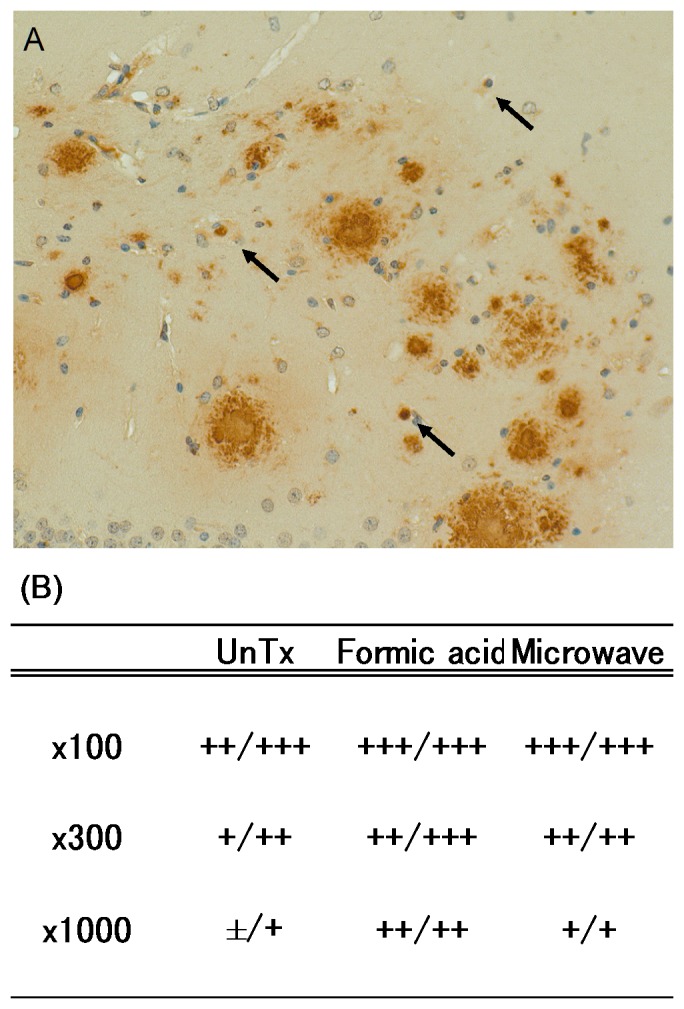
Tissue Amyloid Plaque Immunoreactivity (TAPIR) Assay. (**A**) Paraffin-embedded brains sections of Tg mice were incubated with plasma taken from animals that had been immunized with YM3711. Samples were diluted 1∶100 to 1∶1,000. After washing, sections were incubated with cy3-conjugated or biotinylated secondary antibodies followed by HRP-labeled Vestastain Elite ABC kit. Intracellular Aβ immunoreactivities are indicated by arrows. Brain sections taken from five Tg mice were stained and the representative results are shown. (**B**) Immunoreactivity scores were graded into the following categories: absent immunoreactivity, (−); weak immunoreactivity, (+); moderate immunoreactivity, (++); and strong immunoreactivity, (+++).

Findings obtained so far strongly suggest that antibodies induced by YM3711 vaccination direct largely at the conformational epitope of the Aβ complex (oligomers, fibrils and plaques). To confirm this possibility, we performed the tissue amyloid plaque immunoreactivity (TAPIR) assay. As shown in Fig. 8A, YM3711-induced antibodies stained Aβ plaques positively. Interestingly, the antibodies also stained intracellular Aβ (indicated by arrows in Fig. 8A). When we performed TAPIR assay, sections were pretreated with formic acid or microwaving or left untreated. Fig. 8B summarizes the results. First, untreated sections were stained positively, suggesting that the antibodies recognize the surface structure of the Aβ complex. Second, microwave pretreatment which was reported to enhance intracellular Aβ staining [16] work well in this assay. Absorption of plasma taken from YM3711-vaccinated mice resulted in loss of specific staining (data not shown). These findings suggest that antibodies raised by YM3711 vaccination are able to access both extracellular and intracellular Aβ deposits.

### Demonstration of the Absence of T-cell Infiltration in the Brains of Tg and Wild-type Mice Treated with YM3711

**Figure 9:**
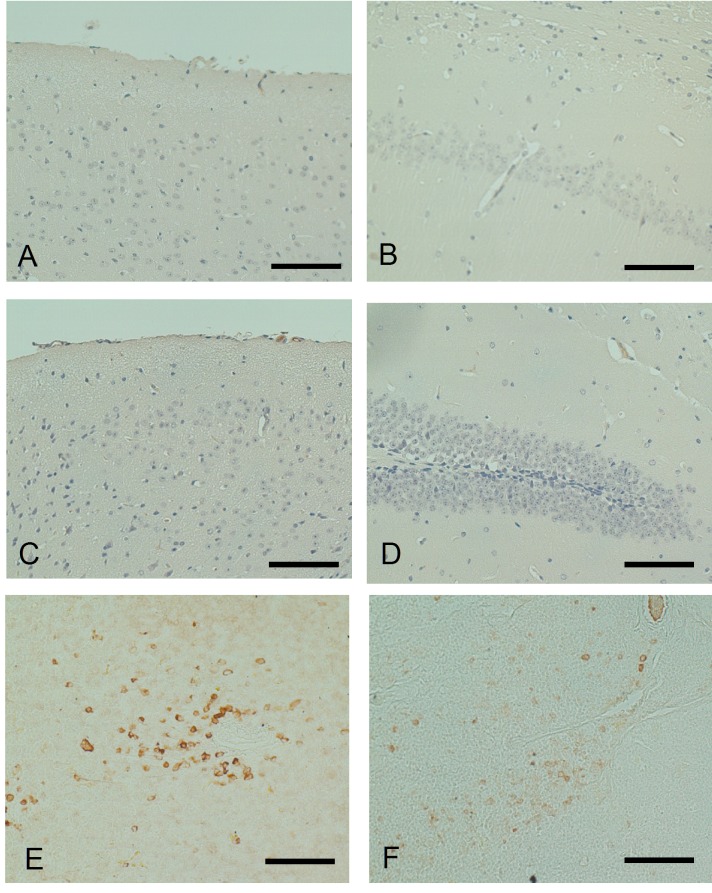
Immunohistochemical examination of the presence or absence of infiltrating T cells in the brains of YM3711-vaccinated Tg mice. Tg and wild-type mice that had been immunized six times with YM3711 were examined immunohistochemically. The number of mice examined is as follows; 18 Tg mice and 4 wild-type mice. Three slices of each cerebrum were sectioned after paraffin embedding. Careful examination of the cerebrum including the cerebral cortex (**A**) and the hippocampus (**B**) of vaccinated Tg mice revealed no infiltrating T cells. Similarly, there was no T cell infiltration in the brain of wild-type mice (**C** and **D**). In the spleen and thymus that had been co-stained as positive control, there were many CD5-positive cells (**E** and **F**).

Finally, we examined the presence or absence of adverse effects of DNA vaccination such as neuroinflammation and microhemorrhage. Careful examination of the cerebrum including the cerebral cortex (Fig. 9A) and the hippocampus (Fig. 9B) of vaccinated Tg mice revealed no infiltrating T cells. Similarly, there was no T cell infiltration in the brain of wild-type mice (Fig. 9C and 9D). In the spleen and thymus that had been co-stained as positive control, there were many CD5-positive cells (Fig. 9E and 9F). These findings indicate that repeated YM3711 administration did not induce neuroinflammation in treated mice. There was no microhemorrhage in brains of vaccinated mice (data not shown).

## Discussion

Anti-Aβ immunotherapies have been developed as a promising therapeutic tool for treatment of AD (reviewed in [17]). Although the first trial with an Aβ peptide vaccine, AN1792, was halted due to the development of meningoencephalitis in some treated patients, autopsy performed later revealed a significant reduction of Aβ plaques compared with unimmunized patients [9]. However, Holms et al. later reported that although AN1792 immunization resulted in clearance of Aβ plaques, this clearance did not prevent progressive neurodegeneration [10]. Since these studies were performed in a relatively small scale and the number of autopsied patients was too small, it is essential to obtain more information to draw final conclusion. Recently, it was reported that AN1792 immunization provided beneficial effects, not only on Aβ pathology, but also on neurite morphology and tau pathology [11]. Furthermore, clinical trials with a humanized anti-Aβ monoclonal antibody, bapineuzumab, revealed that the treatment improved cognitive decline and retarded the brain volume loss in APOE4 non-carrier patients [12]. Collectively, it is essential to start anti-Aβ therapies at a very early stage, even before the mild cognitive impairment stage, as discussed elsewhere [18], [19].

It has recently become recognized that AD is a “conformational disease.” In other words, several Aβ species and amyloidogenic peptides are involved in the development and progression of AD. Interestingly, these molecules, including Aβ1-40, Aβ9-42, Aβ17-40/42, ABri and ADan, form amyloid ion channels that destabilize cellular ionic homeostasis and induce cell degeneration in amyloid diseases [20], [21]. Although, to our knowledge, there is no report on whether AβpE3-42 forms ion channels, it is highly possible that this is the case, as in other pyroglutamated molecules, such as ABri and ADan. Interestingly, in model mice, AβpE plaques increase with age, while the density of other Aβ plaques decreases with aging [22]. This finding suggests that AβpE plaques are more resistant to age-dependent degradation than other plaques. Inhibition of glutaminyl cyclase that mediates N-terminal pE formation attenuates AD pathology [15]. Therefore, it is essential to reduce not only Aβ1-40/42 and their oligomers, but also other Aβ species and amyloidogenic peptides by immunotherapies to obtain beneficial effects on AD.

There have been relatively few attempts to inhibit Aβ aggregation or to develop immunotherapies targeting abnormal protein conformation. Tjernberg et al. first found that a short Aβ fragment can bind full-length Aβ and prevent its assembly into amyloid fibrils [23]. Later, it was demonstrated that several short peptides, termed as β-sheet breakers, inhibited β-sheet formation [24], [25]. Recently, immunization with polymerized British amyloidosis (ABri) related peptide, which has no amino acid sequence homology to Aβ, was shown to reduce Aβ burden and improved cognitive decline in Alzheimer model mice [13].

In the present study, we have developed several new DNA vaccines and extensively characterized the most effective one, YM3711. In vitro studies demonstrated that YM3711 (pVAX-IgL-Aβx4-Fc-IL-4) was transcribed, translated and secreted as one molecule and the added IL-4 sequence exhibited bioactivities. YM3711 effectively induced antibodies in mice, rabbits and monkeys not only to Aβ oligomers, but also to posttranslationally modified Aβ species and other amyloidogenic peptides, which are reported to be neurotoxic [14], [15]. Importantly, vaccination with YM3711 significantly reduced the levels of Aβ1-42, AβpE3-42, Aβ oligomers and Aβ fibrils in Tg mice. Binding and competition assays using YM3711 protein products clearly demonstrated that a large part of antibodies induced by YM3711 vaccination are directed at conformational epitopes of Aβ species and amyloidogenic peptide. Thus, we have successfully produced a DNA vaccine, YM3711 that induces antibodies against a wide variety of Aβ and Aβ species and reduces Aβ deposits without obvious adverse effects.

Very recently, the role of tau in AD became clear and the fyn-tau-amyloid triad seems to be the center of AD pathogenesis [26]. Ittner et al. demonstrated that, using mice expressing truncated tau and tau-deficient mice, tau confers Aβ toxicity at the postsynapse by activating a tyrosine kinase, fyn [27]. It was also shown that Aβ/fyn-induced synaptic, network and cognitive impairments, depending on tau levels [28], [29]. Collectively, phosphorylated tau plays a pivotal role in neurotoxicity but it is less likely that tau shows direct neurotoxic effects. Therefore, Aβ and Aβ-related molecules should be the first target of immunotherapies. However, it is an open question whether anti-tau therapies in conjunction with anti-Aβ therapies are necessary. DNA vaccines have an advantage over other immunotherapies because it is easy to design and modify vaccines in order to add new functions. We are currently preparing several types of Aβ/tau DNA vaccines and will evaluate their efficacies using Aβ/tau model mice.

In summary, we developed a new DNA vaccine (YM3711, IgL-Aβx4-Fc-IL-4) and found that it induced antibodies against a wide variety of Aβ species and other amyloidogenic peptides, resulting in significant reduction of these molecules in the brain of AD model mice. Thus, YM3711 vaccine is worth further examinations in preclinical and clinical trials.

## Materials and Methods

Unless otherwise indicated, all the reagents and equipment were obtained in Tokyo, Japan.

### Ethic Statement

All animal experiments were conducted in accordance with the Guidelines for the Care and Use of Animals (Tokyo Metropolitan Institute of Medical Science, 2011). Experimental protocols were approved by the Animal Use and Care Committee of the Tokyo Metropolitan Institute of Medical Science. Details of animal welfare and steps taken to ameliorate suffering are included in the methods section of the manuscript.

### Animals

B6C3-Tg (APPswe, PSEN1dE9) 85Dbo/J mice (hereafter referred to as Tg mice) were purchased from the Jackson Laboratory (Bar Harbor, ME) and bred in our animal facilities. Tg and wild-type mice at ages between 13 and 15 weeks old were used for the study. Up to six mice were maintained in a cage under conventional conditions. Experimental procedures were performed under isoflurane anesthesia. New Zealand white rabbits were obtained from Japan SLC Inc. Individual rabbits were maintained in a cage. Pentobarbital was used for anesthesia of rabbits. Male (n = 3 for each group) and female (n = 3 for each group) cynomolgus monkeys aged between 3 and 5 years old were used for the study. Ketamine hydrochloride (10 mg/kg) was used for anesthesia of monkeys.

### Animal Conditions

Monkeys were individually housed in stainless steel cages (W750×D750×H700 mm) in an animal room which maintained the temperature at 23±5°C, the relative humidity at 55±25%, the air ventilation at 9 to 15 times per hour and 12-hour illumination per day (07∶00 to 19∶00). Pelleted diet PS for monkeys (Oriental Yeast Co., Ltd.) was given every day (150 g/day) at a set time between 11∶00 and 13∶50 (2 hours after administration or thereafter on the days of administration). The remaining feed was removed in the following morning. Monkeys were allowed free access to drinking water via an automatic water supply system. Examiners frequently accessed to monkeys to maintain good relationship but other enrichment of the monkey’s environment such as toys and periodic access to larger enclosures was not performed.

After the end of the observation period, all animals were euthanized by exsanguination under appropriate anesthesia and subjected to pathological examinations.

For moribund animals and animals that it is judged appropriate to euthanize from the standpoint of animal welfare, the animals will be euthanized and subjected to pathological examinations. However, there were no such animals in this study.

### Development of New DNA Vaccines and *in vitro* Characterization

In a previous study [30], we found that IgL-Aβ and IgL-Aβ-Fc DNA vaccines (listed in Fig. 1A) effectively reduced Aβ deposits in model mice without side effects such as neuroinflammation and microhemorrhages. Based on the results obtained, we produced several vaccines consisting of various combinations of Aβ, Fc and IL-4 to establish safer and more effective vaccine therapy (Fig. 1A). Preliminary and present studies revealed that the IgL-Aβx4-Fc DNA vaccine (Code, YM3711) was most effective.

For in vitro characterization, 4 µg of the indicated DNA vaccine was mixed with 10 µl of Lipofectamine 2000 (Invitrogen) in OPTI-MEM I and the mixture was added to cultured HEK293 cells and the plates were cultured for 4 h. After washing, the cells were further cultured for 3 days. Then, the supernatant and cell pellet of transfected cells were harvested and subjected to Aβ quantitation using the Human β Amyloid (1-42) ELISA Kit *Wako*, High-Sensitive (Wako, Osaka) according to the manufacturer’s instructions. OD_450_ was read with ARVO X3 (PerkinElmer Japan, Kanagawa).

IL-4 activities were determined by bioassay using an IL-4-dependent cell line, TF-1. Culture supernatant of YM-3711-transfected cells or recombinant human IL-4 at various concentrations was added to cultured TF-1 cells. Culture supernatant of non-transfected cells served as negative controls. Using the BrdU uptake assay, the level of bioactive IL-4 was determined. IL-4 activities of the supernatant were determined based on values obtained from serially diluted IL-4.

### DNA Vaccination

For in vivo vaccination, YM3711 at doses of 100 µg for mice, 1 mg for rabbits and 4 mg for monkeys was administered intramuscularly at the indicated time points. Unless otherwise indicated, animals received six biweekly injections. Blood was drawn before and after vaccination and titers of antibodies against the indicated Aβ-related peptides were determined by standard ELISA. At the end of experiments, treated and control animals were killed under deep anesthesia and the brains were removed for examination.

### Immunohistochemistry

Paraformaldehyde-fixed and paraffin-embedded brain sections were stained with mAb (6F/3D) against Aβ8-17 (DAKO). Sections were pretreated in formic acid for 7 min and then incubated in 6F/3D followed by biotinylated horse anti-mouse IgG and horseradish peroxidase (HRP)-labeled Vestastain Elite ABC kit (Vector, Funakoshi). HRP-binding sites were detected in 0.005% diaminobenzidine and 0.01% hydrogen peroxide. For confocal microscopic analysis, FITC or Cy-3 anti-mouse IgG was used as the secondary antibody.

When mouse sections were stained with mouse monoclonal antibodies, the Mouse-On-Mouse (M.O.M.) Detection Kit (Vector) was used according to the manufacturer’s instructions. In brief, sections were incubated in M.O.M. Mouse Ig Blocking Reagent for 60 min and further incubated with 6F/3D or anti-CD5 antibody overnight followed by incubation with M.O.M. Biotinylated anti-mouse IgG and horseradish peroxidase (HRP)-labeled VECTSTAIN Elite ABC Kit (Vector). In order to obtain the optimal conditions for T cell staining, mouse spleen sections were co-stained with anti-CD5.

### Tissue Amyloid Plaque Immunoreactivity (TAPIR) Assay

TAPIR assay was performed according to the methods by Hock et al. [31]. Paraffin-embedded brains sections of Tg mice were incubated with plasma taken from rabbits that had been immunized with YM3711. Samples were diluted 1∶100 to 1∶1,000. After washing, sections were incubated with Cy3-conjugated or biotinylated secondary antibodies followed by HRP-labeled Vestastain Elite ABC kit. For immunofluorescent staining, sections were observed with a confocal microscope (FV1000, Olympus). Immunoreactivities of immunohistochemically stained sections were visualized as described above. Immunoreactivity scores were graded into the following categories: absent immunoreactivity, (−); weak immunoreactivity, (+); moderate immunoreactivity, (++); and strong immunoreactivity, (+++).

### Measurement of Brain Aβ and Anti-Aβ Antibodies in Plasma

For measurement of antibodies against Aβ species and amyloidogenic peptides, microtiter plates were coated with the indicated peptides (2 µg/ml) in 0.1 M sodium carbonate buffer (pH 9.5) for 4 hours at room temperature. After washing, plates were incubated over night at 4°C with serially diluted plasma samples in PBS. The plates were washed and incubated with horse radish peroxidase-conjugated secondary antibodies. Bound antibodies were detected using SIGMA FAST (Sigma-Aldrich) and the absorbance at 450 nm was read on an automated plate reader (Model 550; Bio-Rad laboratories). Titers of anti-human Fc and anti-human IL-4 antibodies were determined in a similar way using human IgG-Fc fragment (Bethyl) and recombinant human IL-4 (Preimmune Inc.) as antigens. To avoid inter-assay variations, all the samples to be compared were assayed at once.

The amount of Aβ and related molecules was quantitated by a sandwich ELISA, Human Amyloid β (1-42, pE3-42 or Oligomers) Immunoassay Kit (IBL, Takasaki, Japan), according to the manufacturer’s instructions. The brain tissue was homogenized in a guanidine-HCl buffer and the supernatant was collected after centrifugation. An appropriate amount of the brain extract was subjected to the assay.

### Western Blot Analysis of Anti-Aβ Oligomer Antibodies in Vaccinated Animals

Lyophilized Aβ1-42 peptide (Peptide Institute, Inc., Osaka, Japan) was suspended in 1 mM HFIP (Hexafluoroisopropanol; Nakalai Tesque, Kyoto, Japan) for 2 h at room temperature. The HFIP was allowed to evaporate in the fume hood and the resulting clear peptide films were dried with nitrogen gas. The HFIP-treated aliquots were resuspended to 5 mM in DMSO (dimethyl sulfoxide; Nakalai Tesque). Aβ oligomers were prepared by diluting Aβ1-42 peptide to 100 µM with phenol red-free RPMI medium (Invitrogen), incubating at 37°C for 24 h.

Soluble Aβ oligomers were also extracted from frozen brain tissue of model mice. The tissue homogenate in TBS/Calbiochem Protease Inhibitor Cocktail Set III (Merck) was centrifuged at 100,000 g for 1 h at 4°C and the supernatant was harvested. After adding NuPAGE LDS sample buffer (Invitrogen), the samples were incubated at 70°C for 10 min and were run on NuPAGE 12% Bis-Tris gel (Invitrogen) [32]. Then, they were transferred to PVDF membrane (Immobilon-P; Millipore, Tokyo, Japan). After blocking with 10% nonfat milk, the blots were incubated with appropriately diluted plasma to be examined at 4°C overnight followed by incubation with Trueblot HRP-conjugated anti-rabbit IgG (eBioscience, San Diego, CA) (1∶1000) for 1 hr. The blots were developed by enhanced chemiluminescence reagents (ECL Plus Western Blotting Detection System, GE Healthcare) according to the manufacturer’s instructions.

### Western Blot Analysis of Aβ Fibrils

Frontal cortex was subjected to analysis. Cortical tissue was homogenized in cold TBS buffer (50 mM Tris-HCl, 0.2 M NaCl, pH7.5) containing a protease inhibitor cocktail (SIGMA). After centrifugation at 100,000×g for 1 h, the pellet was homogenized in TBS buffer containing 1% Triton X-100. Then, the pellet was again homogenized in Guanidine buffer (5 M GuHCl, 50 mM Tris-HCl, pH8.0) and incubated overnight at room temperature. The supernatant obtained after centrifugation was used for Western blotting. The blots were incubated with anti-Aβ fibrils OC (Merk Millipore) (1∶1000) at 4°C overnight followed by incubation with Trueblot HRP-conjugated anti-mouse IgG and chemiluminescence reagents. The image was obtained with an image analyzed (LAS-3000 mini, Fuji Film) and the densities of 56 kDa bands was quantitated using the Image J Software.

### Purification of Translated YM3711 Product (YM3711P) and Anti-YM3711P Antibodies

For YM3711P purification, HEK293 cells were cultured and transfected with YM3711. After 4 days, culture supernatant was harvested and filtered. Since YM3711P contains the Fc portion of immunoglobulin, the product was further purified on a HiTrap Protein G column (GE Healthcare Japan). The eluate at O.D. 280 nm was harvested and strong Aβ immunoreactivities were confirmed using anti-Aβ mAb, 6E10.

Anti-YM3711P antibodies were purified on a HiTrap Protein G column using 0.5 ml post-immune rabbit plasma. They were then biotinylated with Sulfo-NHS-LC Biotin (Pierce).

### Binding and Competition Assays Using YM3711P

YM3711P at concentrations of 2 µg/ml, 10 µg/ml and 20 µg/ml or Aβ1-42 (2 µg/ml) were coated onto microtiter wells. After blocking, biotinylated IgG purified from plasma of rabbits that had been vaccinated with YM3711 were applied and followed by HRP-labeled VECTSTAIN Elite ABC Kit. Bound antibodies were detected using SIGMA FAST (Sigma-Aldrich) and the absorbance at 450 nm was read. Samples showing O.D. more than 2.5 were further diluted and reexamined. Calculated O.D. values are shown in the figure.

For competition assay, YM3711P at a concentration of 2 µg/ml was applied onto microtiter wells. Then, wells were incubated with a mixture of biotinylated anti-YM3711P IgG and unlabeled various competitors at 0.1 to 100 ratios. Competitors included 6E10 (Covance Japan), 4G8 (Covance Japan), anti-AβpE3-42 antibodies (Immuno-Biological Laboratories), anti-Aβ oligomer antibodies (Life Technologies Japan) and unlabeled anti-YM3711P IgG. Then, the absorbance was read in the same way as the binding assay.

### Statistical Analysis

The Student’s t test or Mann-Whitney’s U-test was used for the statistical analysis. P-values less than 0.05 were considered significant.
